# A comparative study of allowable pesticide residue levels on produce in the United States

**DOI:** 10.1186/1744-8603-8-2

**Published:** 2012-01-31

**Authors:** Roni A Neff, Jennifer C Hartle, Linnea I Laestadius, Kathleen Dolan, Anne C Rosenthal, Keeve E Nachman

**Affiliations:** 1 Center for a Livable Future, Johns Hopkins Bloomberg School of Public Health, 615 N. Wolfe St. Suite W7010, Baltimore, MD 21205-2179, USA; 2 Department of Environmental Health Sciences, Johns Hopkins Bloomberg School of Public Health, 624 N. Broadway, Baltimore, MD 21205-2179, USA; 3 Department of Health Policy & Management, Johns Hopkins Bloomberg School of Public Health, 615 N. Wolfe St., Baltimore, MD 21205-2179, USA; 4 Association of State and Territorial Health Officials, 2231 Crystal Drive, Suite 450, Arlington, Virginia 22202, USA; 5 Fulbright Scholar, Center for Studies on Sustainable Development, CP 130/02, 50 av. F. Roosevelt, 1050 Brussels, Belgium

**Keywords:** Pesticides, Agriculture, International Trade, Policy, Food Safety

## Abstract

**Background:**

The U.S. imports a substantial and increasing portion of its fruits and vegetables. The U.S. Food and Drug Administration currently inspects less than one percent of import shipments. While countries exporting to the U.S. are expected to comply with U.S. tolerances, including allowable pesticide residue levels, there is a low rate of import inspections and few other incentives for compliance.

**Methods:**

This analysis estimates the quantity of excess pesticide residue that could enter the U.S. if exporters followed originating country requirements but not U.S. pesticide tolerances, for the top 20 imported produce items based on quantities imported and U.S. consumption levels. Pesticide health effects data are also shown.

**Results:**

The model estimates that for the identified items, 120 439 kg of pesticides in excess of U.S. tolerances could potentially be imported to the U.S., in cases where U.S. regulations are more protective than those of originating countries. This figure is in addition to residues allowed on domestic produce. In the modeling, the top produce item, market, and pesticide of concern were oranges, Chile, and Zeta-Cypermethrin. Pesticides in this review are associated with health effects on 13 body systems, and some are associated with carcinogenic effects.

**Conclusions:**

There is a critical information gap regarding pesticide residues on produce imported to the U.S. Without a more thorough sampling program, it is not possible accurately to characterize risks introduced by produce importation. The scenario presented herein relies on assumptions, and should be considered illustrative. The analysis highlights the need for additional investigation and resources for monitoring, enforcement, and other interventions, to improve import food safety and reduce pesticide exposures in originating countries.

## Background

Since the 1980's, fruit and vegetable consumption has risen across the U.S. About half of the increased demand for fresh fruit and a quarter of the demand for fresh vegetables has been met by imports. Since NAFTA was signed in 1992, fruit and vegetable importation to the U.S. from trade partners has nearly quadrupled [1]. The primary drivers of U.S. consumer demand include the desire to eat off-season and tropical fruit items, promotion of produce-rich diets, and lower prices available from other countries, particularly when supported by favorable terms in trade agreements [1,2]. The U.S. Department of Agriculture indicates that 48.8 percent of fresh fruits and 25 percent of fresh vegetables consumed in the U.S. in 2010 were grown abroad. This reflects a significant increase from 1990, when only 40.4 percent of fresh fruits and 9.9 percent of fresh vegetables were imported (Glaser, L., USDA, personal communication, October 24, 2011). For some produce items, importing is relatively consistent year round; others vary seasonally.

Countries exporting produce to the U.S. are required to adhere to U.S. pesticide tolerance limits, defined as the amount that may legally remain on food post-production. U.S. agencies perform fairly low levels of testing on these imports, as will be described; information thus remains limited on the extent to which these imports may expose U.S. consumers to elevated levels of pesticide residues or other contaminants, relative to domestically grown produce. This analysis aims to gain insight into that gap, and to highlight needs in policy, oversight, and monitoring.

We examine quantities of pesticide residues that could enter the U.S. on top imported produce items, under the scenario that exporters comply with the maximum limits of their own domestic regulations, and not with those of the U.S. In the absence of "negative regulatory feedback" -- frequent inspections and strong enforcement -- there may be little incentive for exporters to comply with U.S. tolerance limits; in some cases, they may even have little awareness of such limits [3]. Accordingly, factors including originating-country regulations might play a stronger role in driving practices and norms than U.S. policy.

There is a complex interplay of factors affecting farmer, intermediary and exporter practice, and affecting pesticide residue levels. It is possible that those exporting to the U.S. fail to comply even with their own nations' residue policies, particularly in developing economies with limited enforcement capacity. Alternately, there is evidence from some countries that farmers may comply with regulations in the top countries to which they export - *in *products for export - but apply lower standards for products for domestic markets [4]. Regardless, farmers are unlikely to apply all allowable pesticides to the maximum limit in all crops, and the level of residues remaining will vary in mixed ways by weather, crop, time of application, and other factors.

### Pesticides and their Health Impacts

Pesticides are defined by the U.S. Environmental Protection Agency (EPA) as "any substance or mixtures of substances intended for preventing, destroying, repelling, or mitigating any pest" [5]. The category includes insecticides, herbicides, and fungicides [5]. Over 20 700 pesticide products were registered for use in the U.S. in 1998, the most recent year for which data are available; these contained approximately 891 active ingredients [6,7]. In 2007, an estimated 498 951 607 kg of pesticides were used in the U.S., and 2 358 680 324 kg were used globally [8]. This estimate includes both synthetic pesticides and other chemicals used as pesticides, such as sulfur and petroleum oil. Residues from these pesticides often remain on or in produce, thus creating a source of human exposure when treated produce items are ingested.

Pesticides work through mechanisms of action intended either to kill pests or render them ineffective [9]. Accordingly, they can also act upon unintended organisms, such as humans. Children in particular may be susceptible to adverse neurological, developmental and other effects from pesticide exposures [6,10]. Despite an extensive literature, the potential adverse health consequences stemming from dietary exposures to a number of routinely-used pesticides remain poorly characterized. Even less well characterized are the cumulative toxicological effects of the complex *mixtures *of pesticide residues and other substances to which we are exposed [11]. Beyond dietary exposures, there are well documented health effects of occupational and residential exposures related to pesticide application to food and other crops. Vulnerable populations including children and pregnant women may face particular risk from such exposures [12,13].

### Pesticide Policy in the U.S. and Abroad

While the U.S. Food and Drug Administration (FDA) is charged with inspecting domestic and imported produce for food safety purposes, the EPA is responsible for monitoring the use of pesticides in domestic food production and establishing limits on the amount of pesticide residue that can remain in or on foods sold. The EPA's pesticide work has traditionally been driven by three main statutes:

1) The Federal Food, Drug, and Cosmetic Act (FFDCA), which establishes maximum residue limits (MRLs) for pesticides on food in interstate commerce, including imports [14];

2) The Federal Insecticide, Fungicide, and Rodenticide Act (FIFRA), which governs the sale and use of pesticide products in the U.S. through registrations. Only these registered pesticides are allowable on imports [15]; and

3) The Food Quality Protection Act (FQPA), passed in 1996, which amended both FFDCA and FIFRA, including mandating a "reasonable certainty of no harm" standard for pesticide safety [16]. It also required that in setting MRLs, the EPA must consider child health, the aggregate risks of exposure to a pesticide in multiple products, and cumulative risks from exposures to multiple pesticides with similar mechanisms of action.

Internationally, many exporters to the U.S. follow either Codex Alimentarius, European Union, or U.S. MRLs. The Codex Alimentarius Commission, established jointly by the United Nations' Food and Agriculture Organization and the World Health Organization in 1963, sets non-binding consensus-based MRLs, as well as other food standards to protect health and ensure fair trade practices [17]. The European Union (E.U.) maintains its own set of standards, which became harmonized across member states in 2008, while Mexico defaults to U.S. MRLs [18,19]. Other countries, including Japan, Canada, Brazil, and Argentina, maintain their own standards. The World Trade Organization's Agreement on the Application of Sanitary and Phytosanitary Measures requires that pesticide standards be based upon "an assessment, as appropriate to the circumstances, of the risks to human, animal or plant life or health" and encourages compliance with the Codex [20,21].

Table 1 illustrates the diversity of MRLs for one commodity across a sample of different pesticides and markets.

**Table 1:** Apple maximum residue limits (MRLS) for selected pesticides and markets, 2008

Pesticide*	U.S. MRL	Codex MRL	E.U. MRL	Canadian MRL
Azinphos-methyl	1.5	2	0.5	2
Inorganic bromide resulting from fumigation	5	20	0.05	---
Metalaxyl	0.2	1	1	1
Methidathion	0.05	0.5	0.05	0.5
Methomyl	1	99**	99**	0.5
Methoxyfenozide	1.5	2	2	1.5
Novaluron	2	3	---	2
Permethrin	0.05	2	0.05	1
Tebuconazole	0.05	0.5	1	---
Thiophanate-methyl	2	3	0.5	5

All units parts per million (ppm).

*Pesticides selected to demonstrate differentials between U.S. and Codex MRLs for apples.

** Established for the sum of methomyl and thiodicarb.

"---" means no MRL for apples was established.

Data: http://www.mrldatabase.com/

### Imported Produce and Pesticide Inspection

The FDA selects import shipments for screening using a "focused sampling" method, which aims to target items at highest risk of exceeding residue tolerances. The FDA may prioritize inspection of specific food types and/or the inspection of imported produce from specific countries based on: past findings; intelligence information; dietary significance; volume imported and domestically produced; and potential pesticide hazards [21]. The FDA's Office of Regulatory Affairs also conducts inspections of domestic and foreign food processing facilities to ensure compliance with the FFDCA [22]. Numbers are low, however; inspections of foreign food processing facilities decreased from 211 inspections in 2001 to 153 in 2008 [23]. The FDA's Office of International Programs has established permanent offices in China, India, Europe, and Latin America in an effort to help build local regulatory capacity and strengthen the FDA's ability to perform timely inspections [24].

According to an analysis by the U.S. Government Accountability Office (GAO), the FDA inspected approximately 1 in every 129 shipments (0.77%) of imported produce during fiscal years 2000-2007. Laboratory analysis was performed on about 1 in 455 shipments (0.22%) [calculated] [25]. In 2009, the FDA employed 272 full time field personnel conducting inspections of imported foods [23]. Current FDA testing protocols cover only about half of pesticides with U.S. MRLs, and exclude many pesticides that are legal internationally but not in the U.S. [21,26]. Further increasing the challenge of detecting violative shipments, importers are known to "shop ports," aiming for ports with lower inspection rates. Overall, FDA capacity to detect instances of excess pesticide residue on imported produce is limited; the agency itself has noted that it neither has, nor will have, "the resources to adequately keep pace with the pressures of globalization" [27].

When FDA tests reveal pesticide residues above EPA tolerances, or residues of pesticides disallowed in the U.S., the entire shipment should be removed from commerce (although this may not always occur) and the relevant exporting companies and originating nations may face an increased likelihood of future scrutiny [28]. An examination of import shipments the U.S. rejected between 1998 and 2004 found that vegetables and vegetable products were the top category of food rejected, with pesticide violations the most frequent cause, reflecting 26.6 percent of rejections of vegetable shipments [calculated] [28].

### Pesticide Residue Monitoring Data

Beyond examining results of enforcement-related screening, we can also gain insights into the relative portion of imports and domestic products with detectable pesticide levels by examining data from governmental monitoring programs aimed at characterizing risks. Two main government programs compile such monitoring data:

The FDA Pesticide Monitoring Program (PMP) annually inspects imported produce at the point of entry to the U.S., as well as domestic produce. In 2008, the agency analyzed 3,656 fruit and vegetable samples [29]. Of these, 4.4% of imported vegetables had violative pesticide residues, compared to 1.7% of domestic vegetables. For fruits, the percentages were 4.8% and 0% respectively (calculated) [29].

The United States Department of Agriculture (USDA) Pesticide Data Program (PDP) conducts tests on agricultural commodities available for consumption in the U.S., with a focus on items commonly consumed by infants and children [29]. In 2009, 9,231 fresh fruit and vegetable samples were tested, covering 14 items [30]. While significant quantities of imports are tested, the 2009 report only presents comparison data on pesticide residues for cucumbers (U.S. vs. Mexico) and grapes (U.S. vs. Chile), and only for pesticides where residues were detected in at least 10 percent of samples. Averaging percent of samples with residue detections for each pesticide, U.S. cucumbers averaged 14.7 percent, compared to 24.1 percent for Mexico. For grapes, the average was 25.6 for U.S. samples, vs. 38.6 percent for Chile [30](Appendix I, calculated). The EPA uses PDP data to calculate a Dietary Risk Index for different commodities, and compares imported and domestic produce items [31].

A broader assessment across markets, produce items and pesticides remains needed.

### Study Purpose

This analysis aims to provide an additional perspective by estimating quantities of pesticide residues to which U.S. consumers could be exposed. Specifically, it models the amount of excess pesticides that could remain on the top U.S. imported produce items if exporters adhered to the maximum allowable application rates based on their own market guidelines rather than those of the U.S. It further aims to present the known health effects associated with low level exposure to the pesticides of concern that we modeled. Finally, it aims to suggest commodity-market-pesticide combinations that should be prioritized for oversight.

This study focuses on examining situations where pesticide residues on imports may pose an excess risk relative to domestic produce, so data on the situations where importation may be protective were not examined in detail. While there may be interest in examining which of these situations dominates, we consider it more useful to consider them separately, each implying a distinct set of concerns and policy responses. The large volumes of produce imports mean significant quantities of excess pesticide residues are introduced into the U.S. food supply, and also suggests environmental and occupational risks internationally, generated by U.S. consumption. Instances where importation could be protective do not "cancel out" these risks. Additionally, modeling these excesses helps identify specific targets for increased scrutiny, thus contributing to the potential for intervention.

## Methods

### Identifying commodities, markets, and pesticides for examination

The analysis relies on the United States Foreign Agricultural Service Maximum Residue Limit (FAS MRL) database for allowable MRLs by commodity, market, and pesticide [32]. This database also indicates which countries subscribe to Codex, European Union or U.S. standards. The database was made public in 2008; it is updated as often as weekly. We downloaded data in August, 2008, and updated data for the E.U. on September 8, 2008 due to widespread changes in standards that had occurred. To prioritize the investigation of potential exposures, the following selections were made:

#### Commodity Selection

Produce items were selected based on a combination of the greatest quantities imported to the U.S., and the greatest overall consumption levels in the U.S. USDA disappearance data, a proxy for consumption, reflects the summed quantities of imports, annual production, and initial stocks of an item minus the total value of exports, non-food uses, and remaining stock of that item per year, on a national level. We used data on retail weight of food disappearance, even though in some cases such as peeled produce, the weight of food as consumed may differ from retail weight. We derived data on both imports and disappearance from the USDA Economic Research Service's (ERS) *Food Availability (Per Capita) Data System *[33]. Fruits and vegetables were ranked by quantities imported and retail disappearance per capita. For each commodity, we summed the two sets of ranks, selecting for analysis the top ten fruits and ten vegetables. (Table 2).

**Table 2:** Top 20 commodities based on import and consumption data, 2007

		Imports, thousand kg (Rank)	Consumption, thousand kg (Rank)	Composite Rank
**Vegetables**	Tomato	1 071 (1)	2 780 (3)	2
	Potato	502 (2)	5 371 (1)	1
	Cucumber	459 (3)	864 (7)	4
	Onion	418 (4)	2 971 (2)	3
	Bell pepper	329 (5)	926 (6)	5
	Squash**	257 (6)	611 (8)	7
	Garlic	225 (7)	405 (9)	9
	Artichoke	166 (8)	221 (10)	10
	Carrot	112 (9)	1 227 (4)	6
	Head lettuce	70 (10)	2 774 (5)	8

**Fruits**	Banana	3 543 (1)	3 552 (2)	1
	Melon	953 (2)	3 851 (1)	1
	Pineapple*	697 (3)	688 (7)	3
	Grape	569 (4)	1 103 (4)	2
	Lime *	328 (5)	326 (10)	6
	Avocado	302 (6)	471 (8)	5
	Apple	173 (7)	2 262 (3)	3
	Orange	112 (8)	1 021 (5)	4
	Pear	86 (9)	425 (9)	8
	Strawberry	72 (10)	882 (6)	7

Items listed in order of combined ranks for imports and consumption. Consumption measured as "Disappearance." Data: http://www.ers.usda.gov/Data/FoodConsumption/FoodAvailSpreadsheets.htm#fruitveg

* In a few cases, imports exceed disappearance due to re-exportation.

**Squash: No breakdowns could be obtained to specify relative quantities imported of summer, winter, and non-specified squashes, to parallel available data in the MRL database.

#### Market Selection

For each commodity, we included all markets (countries) that supplied at least 453 592 kg annually to the U.S. in 2007, as calculated from the USDA FAS trade database^1 ^(Patrick Woodall, personal communication, July 2008) [34].

#### Pesticide Selection

We used all pesticides included in the FAS MRL database for the relevant country/market combinations. The FAS MRL database only includes pesticides with established EPA tolerances, and only shows pesticide standards when the exporting country has them.

#### Health Effects Identification

To identify potential human health effects and corresponding toxicity values, we located risk assessment documents for each included pesticide. We employed a two-tiered search process: first, we examined the EPA's database of pesticide Reregistration Eligibility Decision (RED) documents for updated toxicity assessments. For pesticides first registered after 1984, we consulted the federal database of government regulations and related documents http://www.regulations.gov. We identified the most recent risk assessment containing chronic toxicity evaluations and abstracted toxicity values.

We also abstracted non-cancer chronic toxicity values for pesticides, preferably in the form of chronic population adjusted doses [cPADs], and cancer slope factors, as well as qualitative carcinogenicity classifications. Where cPADs were not listed, we calculated them from non-cancer reference doses (RfDs). The EPA defines the cPAD as the highest dose at which a person could be exposed over the course of a lifetime, with no expected adverse health effects [35]. This dose is intended to correspond to the most sensitive health effect that occurs as a result of exposure to the pesticide of interest (known as the critical effect), and as a result, is believed to be protective against additional health effects that may be expected to occur at higher doses. We considered acute toxicity values, intended to address high-level exposures such as those potentially incurred by pesticide applicators, to be outside of the scope of this effort.

We identified and categorized the critical effects related to the selected pesticides in a framework primarily based on the work of Fox et al (2004) [36]. Categories were: blood, body weight, bone marrow, cardiac, CNS, developmental, endocrine, GI, kidney, liver, mortality, musculoskeletal, ocular, reproductive, respiratory, spleen, and vascular.

### Analysis

For every commodity-market-pesticide combination, we performed calculations to quantify the difference between MRLs in the originating-country market versus U.S. tolerances. We multiplied differences in MRLs by quantity imported, as a way to model the potential increased or decreased quantity of pesticide residue that could be entering the U.S. food supply, above that allowable on domestic produce. We refer to these combinations as "excess residue" (more residue would enter the U.S. because the originating country has a higher MRL than the U.S); and "reduced residue" (less residue would enter the U.S. because the originating country has a lower MRL than the U.S).

To illustrate the MRL analysis process, Chile has an MRL of 5 parts per million (ppm) for the application of the pesticide Ferbam to grapes. The U.S. has a more restrictive MRL of 4 ppm. The U.S. imported 427 601 527 kg of Chilean grapes in 2007. The modeling would thus show that, 427.60 kg of *excess *Ferbam residue could have entered the U.S. food supply. [1 part/million × 427 601 527 kg.] While not discussed here, the model more broadly suggests that a *total *2 138.01 kg of Ferbam residue could enter the U.S. on Chilean grapes. [5 parts/million × 427 601 527 kg]

This model has important sources of both potential overestimation and underestimation of excesses. Overestimation may be caused by the fact that producers do not generally apply all allowable pesticides at once or in the maximum allowed quantities, and that they may be particularly inclined to follow U.S. regulations in products designated for export. A key source of underestimation is the fact that many producers may exceed their own countries' tolerance limits, and may also apply pesticides banned in produce imported to the U.S. and therefore not included in the model used here [3,4]. The inability to include factors such as weather conditions and the length of time that passes between pesticide application and harvesting, both of which influence the actual residue levels on produce, is also a limitation of the model.

## Results

Across the entire sample, the model suggested that a total of 120 439 kg of excess pesticide residue could be imported into the U.S. if every exporter to the U.S. followed their originating country but not U.S. tolerances. This figure is over and above the allowable residue based on domestic U.S. tolerances. Although it is not the focus of this paper, the modeling also suggested that in items for which foreign regulations were more stringent than those in the U.S., importation could reduce 142 408 kg of pesticide residues.

Table 3 shows the top five pesticides, commodities, and markets, by excess residue modeled to be imported into the U.S. The top produce item, market, and pesticide of concern, respectively, were oranges, Chile, and Zeta-Cypermethrin.

**Table 3:** Top 5 pesticides, produce items, markets (based on modeling as described in methods)

	PESTICIDE	MODELED EXCESS RESIDUE (kg)
1	Zeta-Cypermethrin	23 502
2	Inorganic bromide resulting from fumigation	22 719
3	Methomyl	18 888
4	Thiabendazole	8 132
5	Chlorpyrifos	7 589

	**PRODUCE ITEM**	**MODELED EXCESS RESIDUE (kg)**

1	Orange	19 883
2	Cucumber	18 702
3	Apple	16 660
4	Melon	16 632
5	Banana	15 564

	**IMPORTING MARKET**	**MODELED EXCESS RESIDUE (kg)**

1	Chile	24 530
2	Costa Rica	19 980
3	Spain	16 978
4	Netherlands	13 449
5	Guatemala	12 359

Table 4 provides a detailed look at the top commodity/market combinations with the highest modeled pesticide residue levels. For each commodity/market combination, the table shows the top pesticides contributing to the modeled burden.

**Table 4:** Top commodity/market combinations, and key associated pesticides

Commodity and Market	Total Modeled Excess Pesticides Above U.S. MRLs (Kg)	Pesticide (excesses only)	Potential Pesticide Residues in Excess of U.S. MRLs (Kg)***
Oranges from Spain *	15 169	Abamectin	2 262
		Carfentrazone-ethyl	2 260
		Aldicarb	2 256
		Zeta-Cypermethrin	2 255
		Fenamiphos	2 249
		Oxamyl	2 194
		Fosetyl-Al	1 600
Apples from Chile **	14 846	Methomyl	12 079
		Inorganic bromide resulting from fumigation	1 849
Bell peppers from Netherlands *	11 178	Carfentrazone-ethyl	1 600
		Metolachlor	1 600
		S-metolachlor	1 600
		Methomyl	1 598
		Quinoxyfen	1 596
		Acibenzolar-S-methyl	1 585
		Fenamidone	1 585
Cucumbers from Costa Rica **	11 106	Inorganic bromide resulting from fumigation	9 999
		Propamocarb hydrochloride	500
Grapes from Chile **	7 852	Fenhexamid	4 694
		Boscalid	640
		Quinoxyfen	598
Melons from Guatemala **	7 406	Zeta-Cypermethrin	7 082
Cucumbers from Dominican Republic **	5 972	Inorganic bromide resulting from fumigation	5 377
Melons from Honduras **	4 361	Zeta-Cypermethrin	4 170
Bananas from Guatemala **	4 283	Thiabendazole	2 182
		Chlorpyrifos	2 072
Oranges from Italy *	4 086	Abamectin	610
		Carfentrazone-ethyl	609
		Aldicarb	608
		Zeta-Cypermethrin	608
		Fenamiphos	606
		Oxamyl	591
Bananas from Costa Rica **	4 061	Thiabendazole	2 070
		Chlorpyrifos	1 966
Bananas from Ecuador **	3 641	Thiabendazole	1 855
		Chlorpyrifos	1 762
Melons from Costa Rica **	2 644	Zeta-Cypermethrin	2 529
Tomatoes from Netherlands *	2 270	Fosthiazate	508
		Carfentrazone-ethyl	508
		S-metolachlor	508
Bananas from Honduras **	1 891	Thiabendazole	963
		Chlorpyrifos	915
Bell peppers from Spain *	1 597	Carfentrazone-ethyl	229
		Metolachlor	229
		S-metolachlor	229
		Methomyl	229
		Quinoxyfen	228
		Acibenzolar-S-methyl	226
		Fenamidone	226
Cucumbers from Honduras **	1 531	Inorganic bromide resulting from fumigation	1 378
Bananas from Colombia **	1 478	Thiabendazole	753
		Chlorpyrifos	715
Squash from Costa Rica **	1 340	Methomyl	659
		Zeta-Cypermethrin	659
Melons from Panama **	1 276	Zeta-Cypermethrin	1 221

* EU

** Codex

*** Reflects only pesticides modeled to be imported at levels greater than 1000 lb [454 kg] excess. (In case of bell peppers from Spain, no one pesticide was modeled at > 1000 l b so pesticides at lower levels are reported.)

Examining Table 4 it is notable that all of the produce items with modeled residues high enough to be included in the table originated in markets governed by E.U. or Codex rules. Possible explanations will be considered in the discussion section. The table also highlights the divergence in pesticide standards present even between developed nations.

Table 5 shows the top twenty pesticides based on overall excess pesticide residue, their associated health effects, and the critical effects level identified for each. The table notes the health effects corresponding to chronic, low level dietary exposures to these pesticides, with cPADs listed in corresponding health effect columns. A lower cPAD indicates that a lesser exposure is needed to induce a toxic effect. Additionally, higher levels of exposure or exposures to multiple pesticides may result in additional health effects not noted here. We reviewed toxicity assessments for the top 100 pesticides (by kg excess), finding that body weight and liver effects were the most common health effect categories. Effects on blood, endocrine system, kidneys and central nervous system were also found for at least fifteen pesticides each, while reproductive and developmental endpoints were of concern for seven pesticides each. We do not note carcinogenicity status in the table, as only one among the top twenty pesticides was identified as a probable or known human carcinogen (Captan is a B2 probable human carcinogen). Sixteen of the top 100 pesticides, however, were identified either as known or probable human carcinogens. It should be noted that pesticide toxicity assessments, depending on date, use slightly varying terminology to classify carcinogenicity status. Our approach to classifying for the purpose of this manuscript was to highlight either known or probable human carcinogens.

**Table 5:** Top 20 pesticides and their sensitive health effects ("critical effects")

Pesticide	Excess (kg)	Body Weight^1^	Liver^1^	Blood^1^	Endocrine^1^	Kidney^1^	CNS^1^	Reproductive^1^	Developmental^1^
Zeta-Cypermethrin	10 683	0.06					0.06		
Inorganic bromide	10 327								
Methomyl	8 586					X			
Thiabendazole	3 696	0.10	0.10						
Chlorpyrifos	3 450			0.0003					
Carfentrazone-ethyl	2 468		0.03						
Fenhexamid	2 167			0.17	0.17				
Fenamiphos	1 324			0.0001					
Oxamyl	1 307								
Abamectin	1 307	0.0004							0.0004
Aldicarb	1 302								
Fosetyl-Al	1 201							2.50	
S-metolachlor	1 137	0.10							
Quinoxyfen	1 136	0.20				0.20			
Metolachlor	860	0.10							
Propamocarb hydrochloride	853	X							
Acibenzolar-S-methyl	852			0.25					
Fenamidone	852				0.0283				
Ferbam	653	0.015	0.015	0.015					
Captan	649	0.13							0.13

This table shows the top 20 pesticides modeled to have the highest excess levels when summed across all importing markets. For each, the table shows "critical effects," meaning the most sensitive non-cancer endpoint/s documented in EPA risk assessments.

^1 ^*Numbers in health effects column indicate the chronic population adjusted doses (cPADs) EPA identified, i.e. the highest dose to which a person could be exposed over the course of a lifetime with no expected adverse health effects. cPADs provided are provided in units of mg pesticide per kg bodyweight per day. Value of "X" in health effects column indicates that EPA did not quantitatively estimate a cPAD*.

## Discussion & Conclusions

### Summary

This analysis models the quantity of excess pesticide residues that might be imported into the U.S. assuming exporters comply with originating country tolerances but not U.S. MRLs. The modeling focused on the top 20 produce items based on importation and consumption (disappearance) in the U.S. The model estimates that among those items, and based on the top markets exporting those items and top pesticides used, a total of over 120 202 kg of pesticides *in excess of *U.S. tolerances would enter the country annually, in addition to the maximum levels allowable on domestic produce.

Based on low level dietary exposures, the selected pesticides in this review are associated with health effects on thirteen organ systems, and several are associated with carcinogenic effects. These risks may be of special concern for children, given the prevalence of these foods in their diets, and their smaller size and heightened vulnerability to chemical exposure as a result of developmental processes. It is also critical to note that there are common targets of action for many of the modeled pesticides (Table 6). We emphasize, however, that the pesticide health effects are presented for context only. The analysis lacks data to suggest the extent to which these might be experienced in the population due to food imports.

**Table 6:** Common targets of action for pesticides included in this analysis

Organ System	# of pesticides for which critical effect corresponds to organ system
Body Weight	31
Liver	30
Blood	23
Endocrine	19
Kidney	15
CNS	15
Reproductive	7
Developmental	7
Spleen	4
GI	3
Ocular	3
Respiratory	2
Vascular	2
Unspecified	1
Carcinogens	16

Targets of action were identified based on EPA cpad.

### Methods: Modeling

By its nature, this analysis cannot provide insight into real-world practice; rather, its aim is to model the differences in potential residues based on U.S. and exporting country law. There are no available comprehensive data on the extent to which exporting countries comply with either their own or U.S. MRLs.

Many producers apply pesticides in excess of their domestic tolerances, and some also apply pesticides that are disallowed in the U.S. [3,4] Intermediaries and exporters may also fail to assure compliance. If farmers are not following their own domestic MRLs, the volume of pesticide residues imported in excess of U.S. MRLs might be higher than anticipated by this modeling. Residues on imported produce could also be lower than predicted. Evidence suggests pesticide use in developing nations may diverge by market orientation, with farmers producing for export markets using less toxic (although still not necessarily safe) pesticides relative to farmers producing for national markets, due in large part to increased regulatory pressure from outside countries [37]. Galt (2009) found that gaps in testing and enforcement caused many Costa Rican farmers to feel unrestricted by domestic pesticide standards when producing for their open national markets [4]. Farmers are also unlikely to use every allowable pesticide to the maximum allowable level on each crop. The model is further unable to take into account factors influencing the actual residue levels on produce, such as weather conditions and the length of time that passes between pesticide application and harvesting.

In the years before the U.S. government had solid data on actual pesticide residues in foods, the National Academies of Sciences and others performed modeling analyses similar in concept to ours, examining potential population pesticide exposures based on allowable rather than actual application rates [37]. Industry advocates and others criticized these methods, because existing residue tests showed far lower residue levels. These analyses, and the risk assessments on which they were based, also often used the assumption that everyone consumed every food at the high end of population consumption [38]. While potentially overstating risk for these reasons, the risk assessments also did not yet take account of vulnerable subgroups and of cumulative risk, thus potentially understating other risks. Improved dietary survey data and Monte Carlo analysis methods eventually enabled more realistic risk assessments, as currently required under the Food Quality Protection Act [16] (although further improvement remains needed).

Modeling analyses based on policy, such as this one, are still needed for several reasons. First, these methods provide a relatively inexpensive way to help identify pesticides, foods, or importing markets of potential concern for further followup. If significant policy gaps are identified for widely consumed foods, the case may be made for additional inspection resources. Second, analyses like this one highlight tolerance policy differentials between markets, which may be key targets for future attention or harmonization. As noted in Table 1 there is substantial divergence in pesticide standards present even between developed nations. Finally, the lack of sufficient import laboratory testing means that data simply do not exist to perform the needed analyses in a more rigorous way at this time.

### Methods: "Excess" vs. "reduced" residues

In this model, importing the top 20 produce items was slightly more likely to be "protective" due to more stringent tolerances in originating countries than in the U.S. for the examined commodity-market-pesticide combinations. However, we emphasize the benefits of considering the "excess" and "reduced" categories separately, as their impacts are not additive. An exposure prevented does not minimize the impact of another exposure that exists.

This analysis focused on the excess residues, for three primary reasons. First, there remains a large volume of excess pesticides imported into the U.S. on produce, with potential public health ramifications in this country. Second, these "excesses" may reflect environmental and occupational health threats affecting exporting countries, generated by global trade. Third, focusing on excess residues is helpful for highlighting products potentially in need of increased governmental screening.

By contrast, a study examining areas where importation is protective might be useful for discussion of questions about the strength of U.S. tolerance assessments relative to those internationally. Indeed, we note that while U.S. tolerances served as the baseline for the model, neither their protectiveness nor domestic levels of compliance with these standards was examined.

### Methods: Exposure and Risk

The model considers potential pesticide exposures and risks in only the most basic way. Exposure is expressed in terms of how much excess residue may be entering the food supply. The analysis does not account for population variation in diet, "dose" or varying food consumption in vulnerable populations. We note that many of the produce items we identified as having the largest excess pesticide residues also have skins or outer shells that consumers do not consume; the removal of skins and shells will significantly reduce pesticide residue exposure risk. These produce items remain important, however, because while most pesticide may be on the skin, some may penetrate skins and/or be absorbed through roots to permeate the item. Dermal exposure is also a potential concern.

Risk too is characterized in a basic way for this model. EPA's approach used to derive cPADs accounts only for the individual impact of a single chemical on a single health endpoint. Many of the studied pesticides, however, when acting alone, can act on multiple organ systems. Even under circumstances when an exposure to a single chemical may be insufficient to elicit an adverse effect, the cumulative impact of multiple pesticides may be additive or synergistic; thus, simultaneous exposure to numerous chemicals may result in toxicological effects. This suggests that existing residue tolerances may be inadequate to protect public health. These concerns exist independently of international differences in pesticide tolerances, and stem from the antiquated approach of evaluating pesticides on a single chemical basis.

### Context: Top Items of Concern Identified by Others

It is useful to consider how the findings from this analysis compare to those from monitoring and other assessments. Table 7 shows the top produce items of concern identified by the FDA Pesticide Monitoring Program, EPA Dietary Risk Index, the nonprofit Environmental Working Group (EWG), and this analysis. EWG annually presents a "dirty dozen" produce items, based on data from both the USDA and FDA testing programs. Their compilation does not create distinct lists of domestic and imported produce, but rather highlights those items it finds to be of most concern regardless of origin.

**Table 7:** Top produce items of concern, 2007

FDA Pesticide Monitoring Program* (imported commodities)	EPA Dietary Risk Index** (imported commodities)	Environmental Working Group's "Dirty Dozen" (imported and domestic commodities)***	Top 10 Items of Concern (based on excess residue modeling as described in methods)
***Fruits and Vegetables***	***Fruits***	***Fruits and Vegetables***	***Fruits and Vegetables***
Berries, dried or paste	Grapes	Peaches	Oranges
Ginseng, herbal and botanical, other than tea	Peaches	Apples	Cucumbers
Snow peas	Cantaloupe	Sweet bell peppers	Apples
Mango, dried or paste	Apples	Celery	Melons
Celery, dried or paste		Nectarines	Bananas
Chinese okra (luffa)		Strawberries	Bell peppers
Chinese/Thai eggplant	***Vegetables***	Cherries	Grapes
Red beet	Lettuce	Kale	Summer Squash
Pear	Peppers	Lettuce	Tomatoes
Chutney	Cucumbers	Grapes (imported)	Winter Squash
Papaya	Celery	Carrots	
Spinach	Tomatoes	Pears	
Blackberries			

*The FDA Pesticide Monitoring Program annually samples imported produce at the point of entry to the U.S.

** The EPA Dietary Risk Index is based on data from the USDA Pesticide Data Program, which conducts tests on agricultural commodities available for consumption in the U.S.

*** Environmental Working Group's "dirty dozen" reflects an analysis of data from both USDA and FDA testing.

As can be seen in Table 7 there is substantial variation in the top produce items of concern. This may be significantly explained by different criteria for inclusion. The FDA list of items of concern may have been driven by particular outlier findings in compliance inspections rather than reflecting the full spectrum of food shipments - a possibility suggested by the relative rarity in the food supply of many of the highlighted items. The EPA Dietary Risk Index (2006) is a function of the percent of positive tests in the USDA's Pesticide Data Program, and the level of potential risk (which includes pesticide toxicity, children's consumption levels, and residue levels.) [31], and thus takes into account a broader range of data; the EPA report only covered a small number of produce items altogether, and did not indicate how these were chosen. The EWG compiles its report based on a composite index score reflecting six measures of pesticide contamination [39]. Of EWG's so-called "dirty dozen," only seven met the criteria to be included in our model (apples, sweet bell peppers, strawberries, lettuce, imported grapes, carrots, and pears) based on quantities imported and importing markets. Further, no data from strawberries impacted our models, because only Mexico exported enough strawberries to the U.S. to be included in the model, and Mexico uses U.S. pesticide tolerances. The findings from all of these different types of analyses should be considered, as inspection and monitoring priorities are determined.

### Context: International Production Practices

The analysis found that the 20 commodity-market combinations with the highest modeled pesticide residues all followed E.U or Codex regulations. Perhaps this finding is to be expected. Wealthier countries such as those in the E.U. may have more pesticides registered than less wealthy countries, because manufacturers may target them for marketing. Even if such countries were more stringent than the U.S. for half the pesticides and less stringent for the other half, that would mean half their pesticides would enter our modeling calculations. With more pesticides counted, as well as high trade volumes, it makes sense that wealthier countries would rate high in this model. Countries following Codex standards may be overrepresented for a different reason: Codex standards are developed through a representative international process, and as such, might be pushed to a lower level than those in the U.S in order to succeed in obtaining broad support [40]. Verifying this possibility is beyond the scope of this analysis.

It is also important to note that the top five markets of concern identified in this analysis include both developing and developed nations. While one might anticipate that developed nations would be more likely to export crops that meet U.S. MRLs even if their own MRL is less stringent, there is evidence that illegally high levels of pesticide residue are not limited to developing nations [41], although the top causal factors underlying violations will vary by economic and other factors. Spain, for example, has a high rate of illegal pesticide residue levels relative to the number of FDA samples taken. Arguably, this is because Spain's primary export market is the E.U., thus reducing the incentive to adapt practices for U.S. markets [41].

There remains little U.S. oversight of how imported produce is grown or of the working conditions that farmers face. While export production may yield economic benefits, it can also mean prime agricultural resources are diverted to producing for export rather than for domestic consumption, with impacts on food security in exporting countries. Further, in order to bolster exports, some farms may turn to monocropping systems and the use of genetically modified seeds that can result in increased pesticide use. Financial pressures arising from low farm prices may also encourage the application of illegal levels of pesticides in order to cut costs and boost production [41]. As one of the larger produce importers in the world, the U.S. bears some responsibility for these negative outcomes.

Proper enforcement of U.S. tolerances could help encourage exporting farmers to limit their pesticide use to prevent produce from being turned away by U.S. inspectors, which could in turn lead to a farmer losing future business with export firms. In developing nations without strong enforcement of domestic MRLs, U.S. tolerances can indeed represent a key means of improving production standards. A number of studies have documented the beneficial impacts that outside standards, when clearly enforced, can have on production practices in developing nations [3,42,43]. It should be noted, however, that farmers may also seek to avoid residue violations through the use of pesticides that break down rapidly rather than pesticides that actually comply with U.S. standards [3]. It should also be stressed that U.S. standards are not necessarily safe even if they do represent an improvement on many developing nation standards, as they too are subject to limitations and political pressures [38]. Finally, given the practice of "pesticide divergence by market orientation," it is unlikely that U.S. standards will impact pesticide use among farmers producing for their own domestic markets [42].

### Strengths and Limitations

The strength of this analysis is that it uses a newly available database to help understand the public health implications of pesticide levels on imported food. As discussed above, limitations include the artificiality of assumptions and the inability to model real world scenarios of pesticide application, produce consumption, and potential health effects. This analysis is not intended to provide such information; rather, it aims to model the differences in potential residues based on U.S. and importing country law. An additional limitation is that we did not verify the quality of the underlying data provided on governmental websites.

### Implications for Policy

These results demonstrate the need for increased efforts to characterize the levels of actual pesticide residues on imported produce that may be entering the U.S. The Food Safety Modernization Act of 2010 (FSMA) represents an important step forward. Under the FSMA, importers of foreign foods (those who own shipments at the time of U.S. entry) must establish foreign supplier verification programs to ensure that the foods they are importing are produced in compliance with specified provisions, including a mandate that producers evaluate and take action to minimize or prevent any reasonably foreseeable hazards from pesticides in use at a facility. The FDA must also establish additional offices in foreign countries to aid agencies there in ensuring the safety of foods exported to the U.S., and may now make agreements with foreign governments regarding inspection of foreign food facilities. The FDA can also refuse shipments from facilities or countries that do not allow inspections. Within the first year of enactment, at least 600 foreign food facilities must be inspected, with that number doubling every year for a period of five years. However, the FDA has noted that this will quickly become impossible without significantly more resources or substantial restructuring of FDA operations [27]. Indeed, the sheer volume of imports to the U.S. suggests that FSMA may not be able to significantly address the challenge at hand, particularly if the legislation is not fully funded.

In June 2011, the FDA declared a new strategic focus on international partnerships and data sharing with foreign agency counterparts in an effort to ensure a safer food supply in the face of limited resources and an ever growing number of imports. As a part of this strategy, the FDA will also focus increasingly on risk analytics and targeting surveillance efforts on risk-based priorities, as well as greater use of public and private third-party auditors [27]. The implications of this strategy for pesticide inspection have yet to be seen, however, it may represent a significant departure from the current model of operations.

Overall, the uncertainties highlighted in this investigation reinforce the message that the U.S. produce import inspection system needs strengthening. Failure to enforce U.S. regulations can mean increased exposures to pesticides not only for U.S. consumers, but also for exporting country farmers and communities where domestic enforcement may be lacking [3]. Following are a set of policy responses that would help address these concerns.

1) Appropriate full funding for the FSMA and ensure that provisions for increased import inspections and international food safety capacity building are fully implemented.

• Continue to improve assessments of exporting country oversight and provide technical assistance and support for improving oversight effectiveness.

• Work to ensure that farmers and exporters have adequate information about U.S. regulations and food production methods that minimize pesticide usage.

• Require unique identifiers for food importers, to enable improved inspection targeting and an improved incentive for importers to ensure regulatory compliance. While the FSMA mandates a study of unique identifiers, the law contains no requirement for their establishment.

2) Improve FDA pesticide inspections and develop additional programs beyond current requirements.

• Increase funding for FDA import inspections and laboratory testing, including pesticide screening.

• Specifically address pesticide inspections in new efforts to promote FDA collaboration with third-party auditors and foreign agency counterparts.

• Strengthen FDA standards governing expectations for the frequency of import shipment screening and laboratory testing. The standards should specify an expanded list of pesticides for screening based on on-the-ground investigation of the most commonly used pesticides for particular items in top exporting markets. On a pilot basis, enhance inspections of the items identified in this modeling.

• When legally appropriate and feasible, destroy tainted shipments to assure that increased inspections do not lead to shipments entering the food supplies of other countries.

• Improve transparency in FDA inspection records, such as by establishing a publicly accessible database.

• Address gaps in Country of Origin Labeling (COOL) policy. The U.S. requires COOL labels on most fruits and vegetables (as well as some other products), but labels are still not required for items such as processed fruits and vegetables, and fruit juices [44]. Such labels can help consumers make informed purchasing choices.

3) Re-examine U.S. tolerances for pesticides where other countries have more stringent requirements.

4) Expand USDA and FDA pesticide residue monitoring programs to include a greater number of samples analyzed annually, with a particular emphasis on imported produce. Develop a cross-agency complementary strategy to avoid duplication.

5) Pause trade negotiations pending improvements in oversight capacity. As described above, a significant portion of the increase in fruit and vegetable imports may relate to the reduced tariffs negotiated through trade agreements. The U.S. inspection system is currently unable to absorb further increases in imports through new trade agreements.

### Conclusion

The U.S. imports a significant and rising portion of its fruits and vegetables, and the impacts of this practice on domestic pesticide exposure have yet to be characterized. Based on the limited extent of pesticide residue screening and the potential for increased exposures, a more rigorous hazard evaluation is warranted. The commodity-market-pesticide combinations identified in this analysis are worthy of attention. Without strict enforcement of pesticide residue regulations, exporters have little reason to meet pesticide tolerance levels for the U.S. or their home country. There is a critical need for increased produce inspection and pesticide residue screening. Filling this information gap is a necessary step to improve food safety.

## Endnote

^1^Calculations were performed initially in lbs rather than kg, and thus the original cutoff value was 1 million lbs.

## Competing interests

The authors declare that they have no competing interests.

## Authors' contributions

All authors read and approved the final manuscript. RN designed most of the study and analysis, managed the project, participated in data management and analysis, and participated in drafting and revising the manuscript. JH participated in data analysis and in drafting and revising the manuscript. LL participated in drafting and revising the manuscript. KD participated in data analysis and in drafting and revising the manuscript. AR participated in data analysis. KN designed parts of the study and analysis, and participated in data analysis and in drafting and revising the manuscript.
